# Attenuation of Polycyclic Aromatic Hydrocarbon (PAH)-Mediated Pulmonary DNA Adducts and Cytochrome P450 (CYP)1B1 by Dietary Antioxidants, Omega-3 Fatty Acids, in Mice

**DOI:** 10.3390/antiox11010119

**Published:** 2022-01-05

**Authors:** Guodong Zhou, Weiwu Jiang, Guobin Xia, Lihua Wang, Molly Richardson, Chun Chu, Bhagavatula Moorthy

**Affiliations:** 1 Institute of Biosciences and Technology, College of Medicine, Texas A&M University, Houston, TX 77030, USA; mbr5500@hotmail.com; 2 Section of Neonatology, Department of Pediatrics, Baylor College of Medicine, Houston, TX 77030, USA; weiwuj@bcm.edu (W.J.); guobin.xia@bcm.edu (G.X.); lihuaw@bcm.edu (L.W.); chunc@bcm.edu (C.C.)

**Keywords:** fish oil, eicosapentaenoic acid, docosahexaenoic, polycyclic aromatic hydrocarbons, DNA adducts, CYP1, DNA repair, detoxification

## Abstract

Numerous human and animal studies have reported positive correlation between carcinogen-DNA adduct levels and cancer occurrence. Therefore, attenuation of DNA adduct levels would be expected to suppress tumorigenesis. In this investigation, we report that the antioxidants omega 3-fatty acids, which are constituents of fish oil (FO), significantly decreased DNA adduct formation by polycyclic aromatic hydrocarbons (PAHs). B6C3F1 male mice were fed an FO or corn oil (CO) diet, or A/J male mice were pre-fed with omega-3 fatty acids eicosapentaenoic acid (EPA) and/or docosahexaenoic acid (DHA). While the B6C3F1 mice were administered two doses of a mixture of seven carcinogenic PAHs including benzo(a)pyrene (BP), the A/J mice were treated i.p. with pure benzo[a]pyrene (BP). Animals were euthanized after 1, 3, or 7 d after PAH treatment. DNA adduct levels were measured by the ^32^P-postlabeling assay. Our results showed that DNA adduct levels in the lungs of mice 7 d after treatment were significantly decreased in the FO or EPA/DHA groups compared with the CO group. Interestingly, both qPCR and Western blot analyses revealed that FO, DHA and EPA/DHA significantly decreased the expression of cytochrome P450 (CYP) 1B1. CYP1B1 plays a critical role in the metabolic activation of BP to DNA-reactive metabolites. qPCR also showed that the expression of some metabolic and DNA repair genes was induced by BP and inhibited by FO or omega-3 fatty acids in liver, but not lung. Our results suggest that a combination of mechanism entailing CYP1B1 inhibition and the modulation of DNA repair genes contribute to the attenuation of PAH-mediated carcinogenesis by omega 3 fatty acids.

## 1. Introduction

A total of 1,898,160 (254,170 for lung and bronchus) new cancer cases and 608,570 (137,040 for lung and bronchus) cancer deaths were predicted to occur in the U.S. in 2021 [1]. Human and animal data have shown that environmental factors (e.g., polycyclic aromatic hydrocarbons (PAHs)) from cigarette smoke, air and water pollution contribute to the etiology of cancers [2,3]. People living near Superfund sites are also exposed to PAHs [4]. PAHs are ranked to be 9th in the national priority list of toxic substances. Specifically, benzo(a)pyrene (BP) and benzo(b)fluoranthene (BbF) are ranked high (8th and 10th), on this list, and are considered to be contaminants of concern (COC) at Superfund sites. PAHs by themselves are inert, but exhibit their cellular effects through metabolism by cytochrome P450 (CYP) enzymes to reactive metabolites that cause the formation of protein and/or DNA adducts, which are associated with mutagenesis, carcinogenesis, and their toxicities. DNA adducts play important roles in the initiation of carcinogenesis [5,6,7,8,9,10].

An epidemiological study [11] showed significant correlations between elevated levels in blood (white blood cells) of aromatic DNA adducts and lung cancer development for up to thirteen years later. Similar results were obtained in Italy [12,13], Spain [14] and Denmark [15]. Animal studies have also shown positive correlations, which are statistically significant, DNA adduct levels induced by PAHs at initial stages (1–7 days after PAH exposure) and tumor incidence observed 10 months later [5]. Therefore, the attenuation of DNA adduct levels is expected to decrease tumor incidence. Reduction in PAH-DNA adduct levels could lessen DNA mutagenesis, which should in turn diminish or inhibit tumorigenesis [16]. The formation of PAH-DNA adducts can be attenuated by detoxification of parent PAHs. PAH-DNA-bound adducts can also be removed by DNA repair processes [17,18,19,20], and injured cells can be eliminated by cell death including necrosis and apoptosis [21,22,23]. During chronic exposures, DNA damage can readily reach steady-state levels in target tissues. In contrast, failure to repair DNA adducts in essential genes or to remove damaged cells containing such adducts is expected to result in accelerated tumorigenesis.

Natural dietary interventions have beneficial properties, including lesser toxicity and side effects [21]. Dietary interventions could lead to the inhibition of adduct formation through several mechanisms: (i) bio-inactivation of parent chemical carcinogens [22], (ii) the enhancement of DNA repair; and (iii) apoptosis [23,24,25]. They may also inhibit the initiation, promotion and/or progression phases of pre-neoplastic lesions to invasive cancers [26,27]. We previously reported that fish oil (FO), which contains omega-3 fatty acids, significantly lowered the levels of total hepatic DNA adducts [28]. Histologica studies indicated that FO played a hepato-protective role during the initial stages of carcinogenesis. It has been shown that FO inhibits NNK-mediated lung carcinogenesis in the A/J mouse [29]. In addition, DHA induces oxidative DNA damage and apoptosis, and augments chemosensitivity of cancer cells [30].

In this study, we tested the hypothesis that the treatment of mice with omega-3 fatty acids, followed by the administration of PAHs would lead to the attenuation of pulmonary PAH-DNA adducts, and the modulation of CYP1 enzymes and DNA repair genes that contribute mechanistically to the suppression of DNA adducts by FO and its components DHA and EPA.

## 2. Materials and Methods

### 2.1. Chemicals

Benzo(a)pyrene (BP), benz(a)anthracene (BA), chrysene, benzo(b)fluoranthene (BbF), benzo(k)fluoranthene (BkF), and dibenz(a,h)anthracene (DBA) were procured from Sigma-Aldrich (St. Louis, MO, USA). Indeno(1,2,3-c,d)pyrene (IP) was obtained from Absolute Standards (Hamden, CT, USA). Fish oil (from menhaden) was from Sigma-Aldrich (St. Louis, MO, USA). Omega-3 fatty acids eicosapentaenoic acid (EPA) and docosahexaenoic acid (DHA) were from Cayman Chemical (Ann Arbor, MI, USA). Materials for DNA extraction [31,32,33,34], RNA isolation [35], and ^32^postlabeling methods [31,36,37] have been published previously.

### 2.2. Animals

B6C3F1 male mice (4–5 weeks) were purchased from Harlan Sprague Dawley (Houston, TX, USA). A/J mice (8–10 weeks) were procured from The Jackson Laboratory (Bar Harbor, ME, USA).

### 2.3. Diets

FO diet and CO diet were obtained from Dytes Inc. (Bethlehem, PA, USA). All diets contained 15% lipid by weight. The fatty acid compositions of FO and CO were from Dytes Inc., as previously described [28]. In order to achieve essential fatty acid requirements, the FO diet contained 3.5 g of corn oil/100 g of diet [38,39]. To prevent the formation of oxidized lipids, the diets were stored at −20 °C in the dark. The FO contained 1 g/kg γ-tocopherol and 0.025% tertiarybutylhydroquinine (TBHQ) as antioxidants. Food grade corn oil was also supplemented with α-tocopherol, γ-tocopherol and TBHQ to obtain antioxidant levels equivalent to those in the fish oil.

### 2.4. Animal Experiments

B6C3F1 mice have been used extensively in chemical carcinogenesis. This strain of mouse was used in our Superfund studies [5]. The A/J mouse is frequently used as a model for testing of lung carcinogenesis. In this study, both strains were used to observe the effects of omega-3 fatty acids on cancer prevention. All animal studies were conducted according to the federal guidelines for the humane and ethical care and use of animals. The studies were approved by the Institutional Animal Care and Use Committees of Texas A&M University Health Science Center and Baylor College of Medicine.

#### 2.4.1. Animal Experiment 1

Male B6C3F1 mice (4–5 weeks old) received the experimental diet (FO or CO) for 30 days, and animals were randomized into 7 groups of 5 mice each per diet, and then exposed to a mixture of PAHs (higher dose; via i.p. administration) containing BP, 86 µg; BA, 262.5 µg; chrysene, 262.5 µg; BbF, 107.5 µg; BkF, 66.5 µg; DBA, 5.5 µg and IP, 33.5 µg per kg body weight. The concentrations of these PAHs were similar to those in residues extracted from contaminated soils of Superfund sites [5]. The concentrations of lower dose PAHs were decreased by 2.5-fold comoared to the higher dose PAHs mixture. These PAHs were dissolved in DMSO:corn oil (50:50). Animals were maintained in the same diet after treatment and were euthanized by CO_2_ asphyxiation and cervical dislocation at 1, 3, and 7 d after the PAH exposure. Lung tissues were dissected and then flash frozen under liquid nitrogen, and stored at −80 °C until further analyses.

#### 2.4.2. Animal Experiment 2

Male A/J mice (8–10 weeks old, 4–5 mice per group) were pre-treated with FO, eicosapentaenoic acid (EPA) and/or docosahexaeoic acid (DHA) or CO by gavage for 3 days. On day 2, the mice were i.p. treated with BP (25 mg/kg). The mice were euthanized 48 h after BP treatment. The doses of EPA and DHA were calculated based on the amounts of these chemicals in FO diet, i.e., 50 and 100 mg/kg for EPA, 30 and 60 mg/kg for DHA, and combination of EPA (50 mg/kg) and DHA (30 mg/kg). EPA and DHA were first evaporated with nitrogen to remove most of ethanol and then were dissolved in CO. The gavage volume of CO or FO was 6.7 µL/g body weight.

### 2.5. DNA Adduct Analyses

DNA extracrion and ^32^P-postlabeling were conducted as reported previously [5,28]. DNA was isolated as reported earlier [33,34] and stored at −80 °C until analysis by ^32^P-postlabeling [31,40], which entailed the nuclease P1-enhanced bisphosphate version of the postlabeling assay [31] with modifications of chromatographic conditions [40].

Radioactively labeled digests were spotted on to modified PEI-cellulose thin layers and chromatographed overnight (15–16 h) with solvent 1 (2.3 M sodium phosphate, pH 5.75), to purify bulky adducts. The bulky DNA adducts were separated with solvents 2 (3.82 M lithium formate, 6.75 M urea, pH 3.35) and 3 (0.72 M sodium phosphate, 0.45 M Tris-HCl, 7.65 M urea, pH 8.2) in the first and second dimensions, respectively [28]. ^32^P-labeled DNA adducts were visualized by screen-enhanced autoradiography at −80 °C using Kodak XAR-5 film or with the aid of an InstantImager (Packard Instruments) [32]. The levels of DNA adducts were calculated, as previously reported [37]. Statistical analysis was performed using one-way analysis of variance (ANOVA) with multiple comparison by Newman–Keuls test [41].

### 2.6. qPCR Analysis

Gene expression assays were used to determine gene expression alterations for selected CYP1, AhR, and DNA repair genes using TaqMan^TM^ assay kits. Gene-specific probes and primer sets were procured from from Applied Biosystems (Foster City, CA, USA, Cat No. 4331182, 4351372, 4331348). The following TaqMan primers were used: *Cyp1a1* (Mm00487217_m1 CYP1A1), *Cyp1a2* (AIRRLEG CYP1A2 mouse), *Cyp1b1* (Mm01232239_m1 CYP1B1), *Ahr* (Mm00478932_m1 AHR), *Ddb2* (Mm00472175_m1 DDB2), *Neil2* (Mm01310470_m1 NEIL2), *Parp1*(Mm01321084_m1 PARP1), Pnac (Mm05873628_g1 PCNA), *Xpc* (Mm01183434_m1 XPC), and the reference gene 18S (Hs99999901_s1 18S). The assays were conducted according to manufacturer’s protocol of TaqMan^TM^ RNA-to = CT^TM^ 1-step kit (Applied Biosystems, Foster City, CA, USA, Cat No. 4392656) on an ABI PRISMH 7900 HT sequence detection system (Applied Biosystems, Foster City, CA, USA). Quantification was performed using the average 2^−ΔΔCt^ value for each set of quadruplicates, and the average of the biologic replicates was calculated. Mouse GRP78 gene was used as the house-keeping control for quantitative RT-PCR. One-way ANOVA was applied in order to compare the levels of gene expression in the lung and liver among five diet groups.

### 2.7. Western Blot Analysis

Microsomes were obtained from lung and liver tissues by differential centrifugation to analyze the protein expression of CYP1A1/1A2 and CYP1B1. Proteins were detected using CYP1A1 monoclonal antibody that cross-reacts with CYP1A2 (1:1500, procured from Dr. P.E. Thomas, from Rutgers University, Piscataway), CYP1B1 antibody (1:2000) (Invitrogen, Carlsbad, CA, USA, Cat. No.: PA5-95277), or the microsome loading control GRP78 (1:500) (Abcam, Cambridge, UK, Cat. No.: ab21685) followed by blotting with HRP tagged anti-mouse and anti-rabbit secondary antibodies (1:10,000, Bio-Rad laboratories, Hercules, CA, USA). Membranes were washed and developed with clarity Western ECL substrate and the BioRad ChemiDoc Touch with Image Lab software (BioRad Laboratories, Hercules, CA, USA). These analyses were performed in quadruplicates.

## 3. Results

### 3.1. Animal Experiment 1

#### 3.1.1. Diet and Body Weights

B6C3F1 male mice were maintained on FO or CO diet for one month before the treatment of PAH mixture. During the study, the diet consumption was measured every day. No significant differences in diet consumption were noted (*p* > 0.05). At 7 days, body weights were 23.89 ± 0.22 g and 23.86 ± 0.20 g for CO and FO group, and at 28 days, body weights were 29.82 ± 0.31 g, and 30.49 ± 0.26 g, respectively. There were no significant differences in body weights between FO and CO diet.

#### 3.1.2. The Effects of Dietary Fish Oil on Pulmonary PAH-DNA Adducts

**Figure 1:**
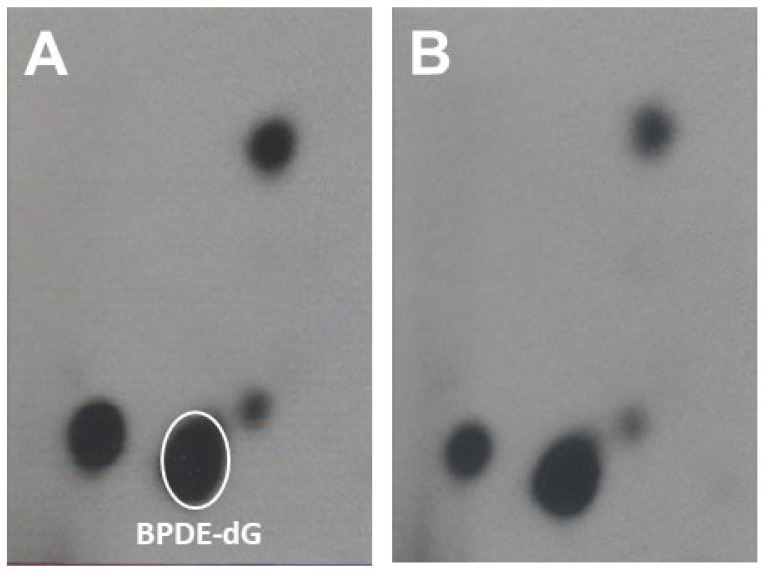
Representative DNA adduct profiles of lungs of B6FC3 mice fed with CO diet (Panel (**A**)) and FO diet (Panel (**B**)) treated Yeswith PAHs mixtures. After one month on a special diet, mice were treated with a high dose of PAHs by a single i.p. injection. BPDE-dG, BP trans-7,8-diol-9,10-epoxide-deoxy-guanosine. Mice were terminated at 7 d after PAHs treatment, and DNA adducts were analyzed by ^32^P-postlabeling, as described under Materials and Methods.

After one month on a special diet, mice were treated with two different doses of PAHs by a single i.p. administration. Mice were euthanized at 1, 3, and 7 d after treatment. Lung DNA from mice on dietary FO or CO displayed qualitatively similar profiles of PAH-DNA adducts with the BPDE-dG being the major adduct in Figure 1.

**Figure 2:**
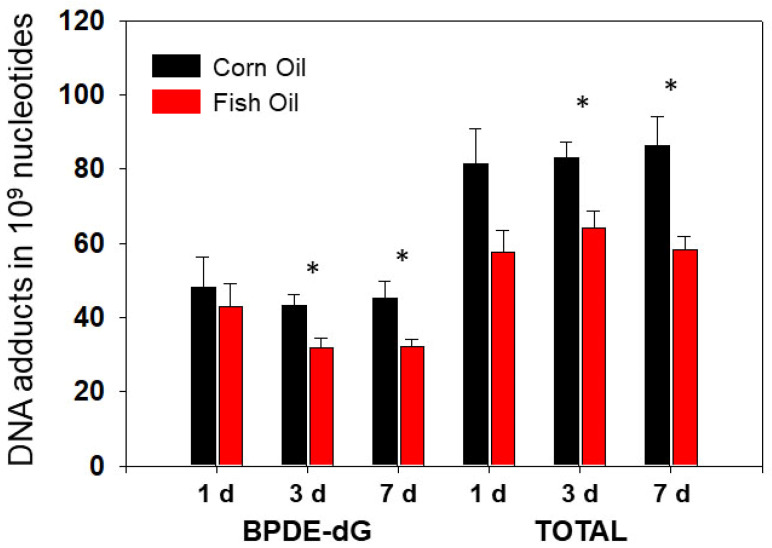
Effect of dietary fish oil on pulmonary DNA adduct levels. Dietary fish oil significantly decreased PAH-DNA adducts compared to CO diet at 3 and 7 days after treatment of high dose of PAH mixture, but not at 1 day. *, *p* < 0.05 (*n* = 4–5, one-way ANOVA).

Lung DNA adducts were formed after treatment with PAH mixture. High levels of PAH DNA adducts were sustained from 1 d until 7 d after PAH treatment. Figure 2 displays the comparisons of the pulmonary DNA adducts of mice treated with a high dose of PAHs between the CO and FO groups. Overall, the levels of total pulmonary DNA adducts were higher in CO groups at all three time points compared with FO groups. However, we observed significant attenuation of PAH-DNA adducts in FO groups compared to those in CO which were only observed at the 3 and 7 d time points, but not at 1 d (Figure 2). The levels of BPDE-dG adducts [42] showed similar trends with total PAH-DNA adducts in both CO and FO groups (Figure 2). After treatment with a low PAH dose, the values of total pulmonary DNA adducts in the CO groups of mice were 38.84 ± 7.70 (mean ± SEM), 54.85 ± 5.51, and 36.35 ± 5.31 in 10^9^ normal nucleotides for 1 d, 3 d, and 7 d, respectively. Correspondingly, these values at FO groups were 37.03 ± 3.30, 42.46 ± 2.90, and 28.70 ± 3.41. Although pulmonary PAH-DNA adduct levels at FO groups displayed trends of lower levels compared to those at CO groups, statistically significant differences were not observed. Similar results of BPDE-dG adduct were obtained (data not shown). Overall, dietary FO decreased the levels of total pulmonary PAH adducts by 22–32%. BPDE-dG adduct displayed similar results.

#### 3.1.3. Histological Examination

Mice did not show significant histological changes in the lungs in both CO and FO groups 7 d after PAH treatment (data not shown).

### 3.2. Animal Experiment 2

#### 3.2.1. The Effects of Omega-3 Fatty Acids EPA and DHA on Pulmonary PAH-DNA Adducts

To further confirm that omega-3 fatty acids played major roles in FO mediated attenuation of carcinogenic PAH-DNA adducts, pure omega-3 fatty acids EPA and DHA were used in a separate animal experiment.

**Figure 3:**
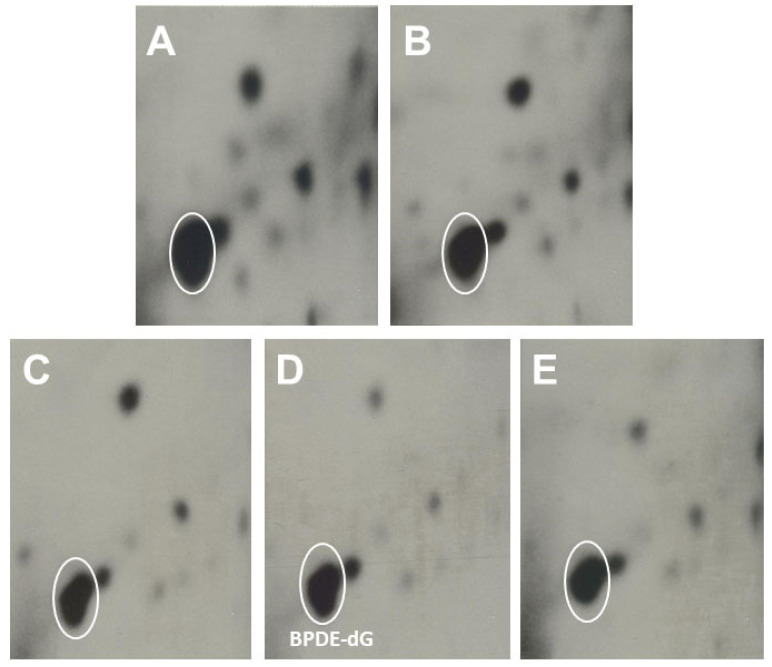
Representative patterns of ^32^P-postlabeled pulmonary DNA adducts in A/J male mice, fed CO, FO, EPA or DHA, treated with BP. Panel (**A**), CO; (**B**), FO, (**C**), EPA (high dose), (**D**), DHA (high dose), and (**E**), EPA + DHA.

Representative profiles of pulmonary DNA adducts in A/J mice, received CO, FO, EPA, DHA, and EPA + DHA, treated with BP, and are shown in Figure 3. The typical patterns of BP-DNA adducts were highly similar in all five groups with only slight differences. As shown in Figure 3, BPDE-dG was the major adduct.

**Figure 4:**
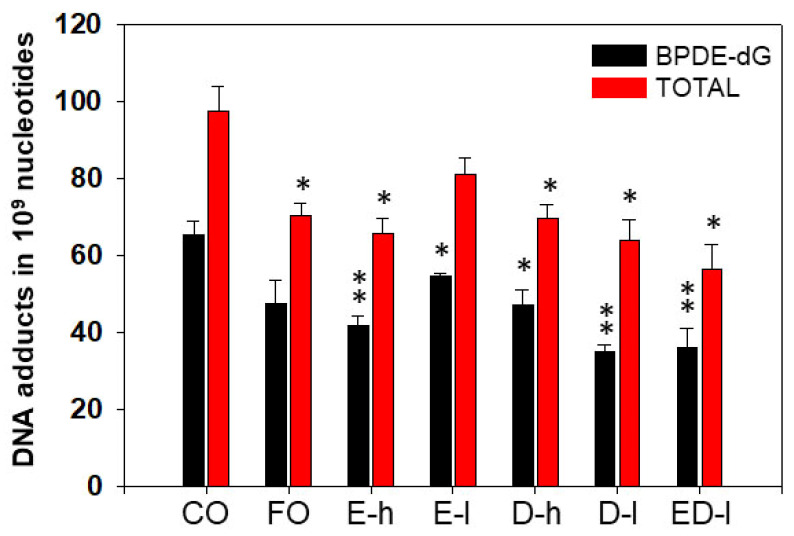
Effect of dietary fish oil and EPA/DHA on pulmonary DNA adduct levels. FO, EPA, and DHA significantly decreased pulmonary BPDE-dG and total DNA adducts in A/J mice compared to CO. *, *p* < 0.05, **, *p* < 0.01; (*n* = 4–5, one-way ANOVA). E, EPA; D, DHA, h, high dose; l, low dose.

The levels of BP-DNA adducts in lung of mice treated with BP were very high in the control (CO) group. However, animal treatments with FO, EPA, or DHA, resulted in significant decreases in total lung BP-DNA adducts and/or BPDE-dG, compared to those in mice that received CO (Figure 4). EPA and DHA, given in combination, displayed more pronounced effects in decreasing levels of total pulmonary DNA adducts and BPDE-dG by 42% and 45%, respectively (Figure 4). FO contains about 30% omega-3 fatty acids, and EPA and DHA are major omega-3 fatty acids in FO. These results suggested that omega-3 fatty acids including EPA and DHA may play key roles in FO-mediated attenuation of PAH-DNA adduct levels.

#### 3.2.2. The Effects of Omega-3 Fatty Acids EPA and DHA on Hepatic PAH-DNA Adducts

**Figure 5:**
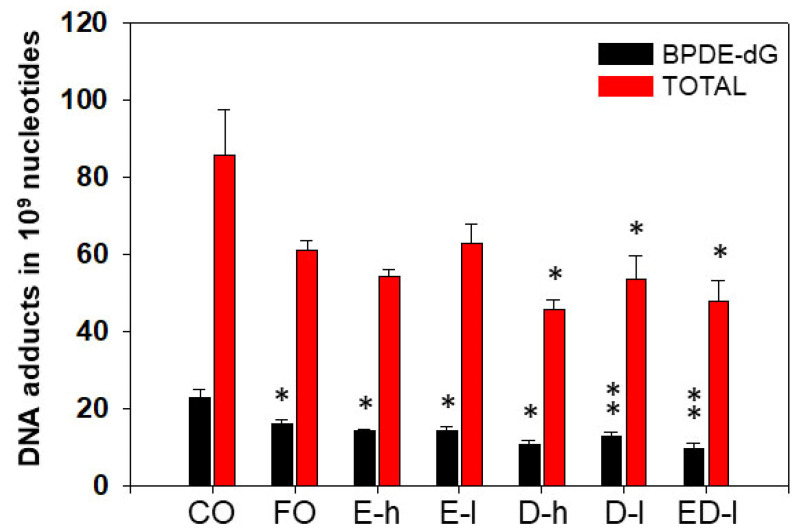
FO, EPA, and DHA significantly decreased liver BPDE-dG and total levels of DNA adducts in A/J mice compared to CO. DHA also significantly decreased hepatic total DNA adducts compared to CO. *, *p* < 0.05; **, *p* < 0.01 (*n* = 4–5, one-way ANOVA). E, EPA; D, DHA, h, high dose; l, low dose.

The levels of hepatic BP-DNA adducts were also determined to further understand BP metabolism and target organs. These results provided us information regarding the mechanisms by which EPA and DHA prevent the liver from PAH-mediated DNA mutation and damage. Overall, BPDE-dG adduct levels in the liver were lower (Figure 5) than those in the lungs, suggesting that the lung is a target organ for carcinogen BP. FO, EPA, DHA, or EPA+DHA significantly suppressed the formation of BPDE-dG adduct compared to CO control with *P*-values being small than 0.05 or 0.01 (Figure 5). The levels of total hepatic DNA adducts were also significantly decreased by DHA or EPA+DHA (Figure 5).

#### 3.2.3. The Modulation of CYP and AHR Genes by BP and Omega-3 Fatty Acids

**Figure 6:**
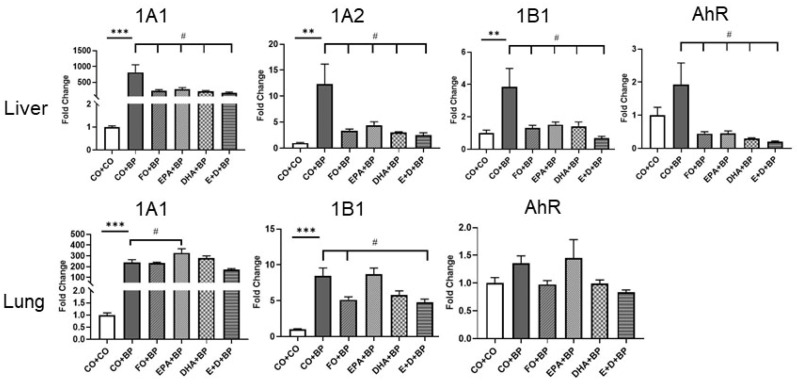
Effects of BP and omega-3 fatty acids on hepatic and pulmonary CYP1, and AhR gene expressions. Real time RT-PCR measurement of *Cyp1a1*, *1a2*, *1b1*, and *Ahr* mRNA expression in liver and lung of A/J mice as written in Materials and Methods. Data are presented as mean ± SEM values by ANOVA analysis (*n* = 5). Significant differences between CO + CO and CO + BP groups are denoted by **, *p* < 0.01, and ***, *p* < 0.001. Significant differences between CO + BP and omega-3 fatty acids + BP are demoted by #, *p* < 0.05.

In order to understand the mechanisms by which omega-3 fatty acids prevent mice from chemical carcinogenesis and the formation of DNA adducts, we analyzed the expression of genes involved in PAH metabolism (e.g., *Cyp1a1*, *1a2*, *1b1*, *Ahr*). As shown in Figure 6, carcinogen BP significantly induced gene expression of *Cyp1a1*, *1a2*, and *1b1* in both the liver and lungs compared to the control (CO). However, omega-3 fatty acids significantly inhibited the expression of these CYP1 genes as well as *Ahr* gene in the liver. Lung *Cyp1a1* and *1b1* were also significantly inhibited by omega-3 fatty acids. The lung’ lacks CYP1A2, so it was not determined. It is well known that CYP1B1 and 1A1 enzymes play pivotal roles in the bio-activation of BP to DNA-reactive metabolites. Inhibition PAH-DNA adducts by omega-3 fatty acids could be detoxification of PAH through the downregulation of CYP1B1 as well as 1A1 mRNA. Our results suggest that EPA and DHA may play a role as nutritional inhibitors of CYP1B1.

#### 3.2.4. Western Blot Analysis

**Figure 7:**
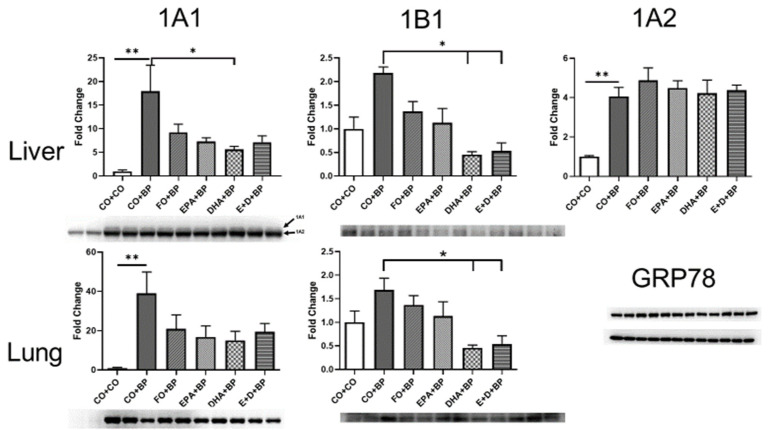
Proteins isolated from liver and lung of mice, fed with CO, FO, EPA, DHA, EPA/DHA and treated with BP, were probed with Cyp1A1, 1B1 or 1A2 (liver only) antibodies by Western blot. FO, DHA and EPA + DHA significantly inhibited Cyp1b1 compared to CO group. Hepatic Cyp1A2 induced by BP treatment was also inhibited by FO, EPA, and DHA. *, *p* < 0.05, **, *p* < 0.01, (*n* = 5).

Western blot analysis for each group showed (Figure 7, Table S1) that the omega-3 fatty acids (EPA and DHA) also significantly attenuated the expression of *Cyp1b1* in both the liver and lungs, as well as *1a1* in the liver only compared to CO. These results further support and confirm the results from gene expression. CYP1B1 expression in the lungs at protein and mRNA levels was induced significantly by PAHs but was suppressed by about 50–70% in the lungs of EPA/DHA-treated mice. This study entailed using at least 4 individual mice per group, and statistical comparisons were performed by one or two-way ANOVA, followed by modified *t*-tests. Our results suggest that EPA and DHA may play a role as nutritional inhibitors of CYP1B1.

#### 3.2.5. The Modulation of DNA Repair Genes

**Figure 8:**
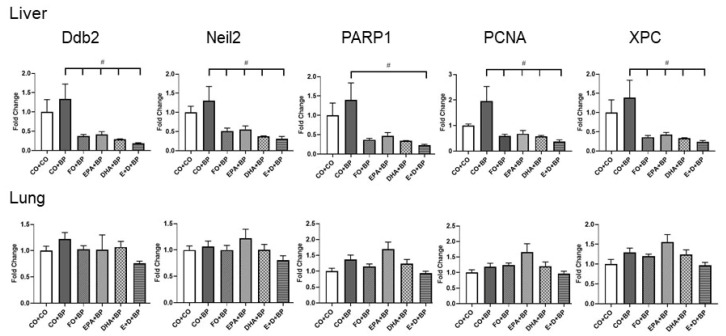
Effects of BP and omega-3 fatty acids on liver and pulmonary DNA repair gene expressions. Quantitative polymerase chain reaction measurement of *Ddb2*, *Neil2*, *Parp1*, *Pcna*, and *Xpc* mRNA expression in lung of A/J mice as stated in Materials and Methods. Data are shown as mean ± SEM values by Anova analysis. Significant differences between CO+BP and omega-3 fatty acids + BP are indicated by #, *p* < 0.05 (*n* = 5).

Since the DNA repair system could play an important role in removing adducted nucleotides by nucleotide excision repair (NER) or base excision repair (BER), we analyzed several genes that are involved in DNA repair. We observed that the expression of *Ddb2*, *Neil2*, *Parp1*, *Pcna*, and *Xpc* genes were significantly inhibited by omega-3 fatty acids in liver but not in lung (Figure 8) compared to CO group. The lungs are a target organ for PAH exposure and there was more DNA adduct formation in the lungs compared to the liver. These results suggested that DNA repair genes show higher expression when DNA is damaged, such as the formation of DNA adducts. High levels of the expression of DNA repair genes will help to remove DNA adducts and prevent from DNA mutation during replications. Eventually, the attenuation of DNA damage and PAH-DNA adducts will reduce the risk of tumor formation.

## 4. Discussion

PAHs including BP are carcinogenic environmental pollutants to which humans are exposed daily. Epidemiological and basic scientific studies have shown that most cancers, including lung cancers, are caused by environmental exposure to complex mixtures (e.g., PAHs from cigarette smoke, PM 2.5, and through diet [43]). DNA adducts are considered biomarkers for carcinogenic PAH exposure in many organs of human [20,44], including smokers [6]. Experimental animal and epidemiological studies have shown that the levels of carcinogen DNA adducts correlated with the incidence of tumors in rodents [5,45] and humans [11,14,15]. Therefore, it is imperative that approaches are developed that can decrease the formation of carcinogen-DNA adducts, repair adducted nucleotides, and remove damaged cells caused by DNA adducts. These effective strategies are expected to significantly decrease lung cancer risk, especially in high-risk populations such as cigarette smokers and in people subjected to occupational exposures.

Several reports have demonstrated omega-3 fatty acids to decrease cancer risk [46]. It has been reported [47] that omega-3 polyunsaturated fatty acids in FO show a variety of health benefits and inhibit the development of human lung and other cancers. Recently, Vega et al. [48] reported that omega-3 fatty acids and their metabolites are thought to alter pivotal pathways underlying the progression of lung cancer. We previously reported that dietary FO decreased hepatic PAH-DNA adduct levels by about 45% in the mice treated with complex PAHs [28]. These observations suggest that such omega-3 fatty acids could act as chemopreventive agents and antioxidants to reduce the liver cancer risk in people exposed to PAH carcinogens [49].

In current studies, we also confirmed that dietary FO and pure omega-3 fatty acids EPA and DHA significantly diminished the formation of pulmonary PAH-DNA adducts. We recently reported [5] there are mechanistic relationships between hepatic PAH-DNA adducts and tumor incidence in B6C3F1 mice treated with PAH mixtures. It is expected that attenuation of PAH-DNA adducts will play a key role in cancer prevention. Menhaden FO contains approximately 30.5% omega-3 polyunsaturated fatty acids (PUFA) including EPA (12.9%) and DHA (8.19%) [29]. Several studies have shown that omega-3 fatty acids play very significant roles in cancer prevention [22,50,51,52] through increasing apoptosis [53,54,55], and by inhibiting cell proliferation [29,56].

In order to further confirm that omega-3 fatty acids play an important role in FO-mediated inhibition of PAH-DNA adduct formation and potential prevention of chemical carcinogenesis, we performed studies with pure omega-3 fatty acids EPA and/or DHA studies. Short-term (3 days) intakes of EPA and/or DHA significantly decreased levels of carcinogen-DNA adducts in the lungs (Figure 3) and the liver (Figure 4) of mice treated with BP. Interestingly, we discovered that EPA and/or DHA significantly inhibited the expression of genes and proteins involved in PAH metabolism such as CYP1A1, 1B1, 1A2, and ARH by qPCR (Figure 5) and Western blot analysis (Figure 7). These results could partially explain the mechanisms how omega-3 fatty acids suppressed the formation of PAH-DNA adducts. It is well known that PAHs are metabolically activated through CYP1B1 [57,58]. CYP1B1 plays an important role in the metabolic activation of PAHs to DNA-reactive metabolites [59]. The inhibition of CYP1B1 could detoxify carcinogenic PAHs, block the formation of PAH-DNA adducts, and suppress tumor formation.

PAH carcinogens have the ability to elicit the formation of DNA damage through a variety of mechanisms, including covalent binding of PAHs with DNA, and oxidative DNA damage [20,60]. It was reported that BP significantly augmented the levels of 8-hydroxy-2′-deoxyguanosine (8-OHdG) and BPDE-DNA adducts in many organs [61]. Although this study did not determine the oxidative DNA lesions by PAHs, and inhibition of oxidative DNA damage by FO, EPA, and DHA, many studies showed that EPA and DHA can decrease oxidative stress and inflammation by acting as antioxidants [62,63]. Wiest et al. reported that EPA and DHA guard against cigarette smoke-induced oxidative stress [64].

Omega-3 fatty acids may play multiple roles in the protection of DNA from damage induced by carcinogenic PAHs. One of the mechanisms is DNA repair pathways which are able to remove mutated or adducted nucleotides [65,66]. DNA repair mechanisms elicit genomic stability by removing DNA damage induced prior to replication completion [67]. In our short-term study, we found that five DNA repair genes were inhibited by omega-3 fatty acids in liver but not in lung. DNA damage including DNA adducts could lead to the augmentation of the expression of genes involved in DNA repair, but this could have happened at a later time point. Consistent with this idea, we observed an increased trend in the expression of pulmonary DNA repair genes in mice exposed to omega-3 fatty acids, especially EPA (Figure 6). Long-term animal experiments that are in progress would confirm these mechanisms.

**Figure 9:**
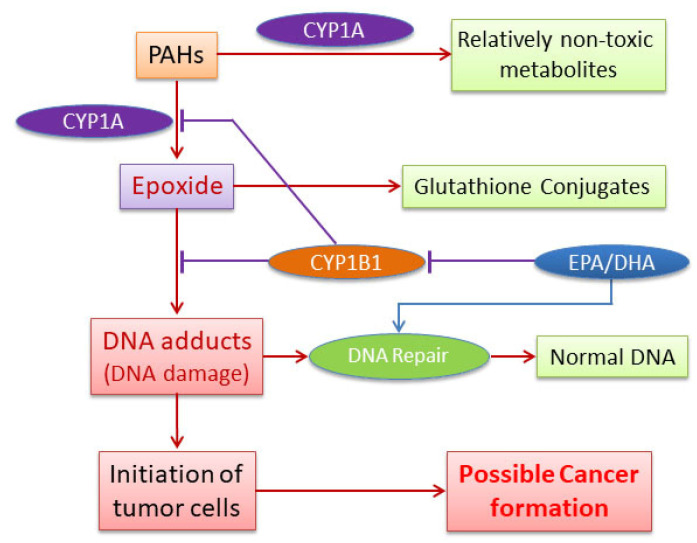
Proposed mechanisms of attenuation of PAH-DNA adducts and tumor formation by omega-3 fatty acids EPA/DHA.

The possible mechanisms of the attenuation of PAH-DNA adducts and the potential decrease in the risk of tumor formation in mice by omega-3 fatty acids are displayed in Figure 9. It was reported that CYP1A1 played important roles in detoxification of carcinogenic PAHs rather than in metabolic activation [68,69,70]. Therefore, the conversion of carcinogenic PAHs to relatively non-toxic chemicals by enzymes will significantly decrease the formation of DNA adducts. Alternatively, omega-3 fatty acids may enhance DNA repair and induce apoptosis when DNA or cells are damaged by carcinogenic PAHs. Omega-3 fatty acids were shown to attenuate cell growth [71] and enhance apoptosis in many human cancer cells [51] including colon [72], prostate [73], pancreas [74], lung [29], and mammary cancers [75]. We have also shown [23] that retinoic acid suppresses BP-adducts in HepG2 cells by inducing apoptosis. However, FO, EPA, an DHA did not cause apoptosis in our current experiments.

As one of the natural chemopreventive agents, FO containing omega-3 fatty acids have attracted significant attention in cancer prevention because of the various health benefits and lesser side effects [21]. The prophylatic actions of chemopreventive agents are mediated via detoxification, DNA repair, and apoptosis. Different natural products may act differently in regard to chemical carcinogenesis. Therefore, novel approaches are needed to develop highly effective chemopreventative agents.

## 5. Conclusions

Our results revealed that omega-3 fatty acids significantly attenuated levels of PAH-DNA adducts in lungs of mice exposed to carcinogenic PAHs. FO, EPA and DHA significantly inhibited expression of CYP1B1. Further research should result in the development of rational strategies (e.g., supplemental FO intake for cancer prevention).

## Data Availability

The data presented in this study are available in this manuscript.

## Supplementary Materials

The following are available online at https://www.mdpi.com/article/10.3390/antiox11010119/s1.

## References

[1] Siegel R.L., Miller K.D., Fuchs H.E., Jemal A. (2021). Cancer Statistics, 2021. CA A Cancer J. Clin..

[2] Grant W.B. (2009). Air pollution in relation to U.S. cancer mortality rates: An ecological study; likely role of carbonaceous aerosols and polycyclic aromatic hydrocarbons. Anticancer Res..

[3] Petit P., Maitre A., Persoons R., Bicout D.J. (2019). Lung cancer risk assessment for workers exposed to polycyclic aromatic hydrocarbons in various industries. Environ. Int..

[4] Suter M.A., Aagaard K.M., Coarfa C., Robertson M., Zhou G., Jackson B.P., Thompson D., Putluri V., Putluri N., Hagan J. (2019). Association between elevated placental polycyclic aromatic hydrocarbons (PAHs) and PAH-DNA adducts from Superfund sites in Harris County, and increased risk of preterm birth (PTB). Biochem. Biophys. Res. Commun..

[5] Phillips T.D., Richardson M., Cheng Y.S., He L., McDonald T.J., Cizmas L.H., Safe S.H., Donnelly K.C., Wang F., Moorthy B. (2015). Mechanistic relationships between hepatic genotoxicity and carcinogenicity in male B6C3F1 mice treated with polycyclic aromatic hydrocarbon mixtures. Arch. Toxicol..

[6] Hecht S.S. (2003). Tobacco carcinogens, their biomarkers and tobacco-induced cancer. Nat. Rev..

[7] Wogan G.N., Hecht S.S., Felton J.S., Conney A.H., Loeb L.A. (2004). Environmental and chemical carcinogenesis. Semin. Cancer Biol..

[8] Hecht S.S. (2012). Lung carcinogenesis by tobacco smoke. Int. J. Cancer.

[9] Stading R., Gastelum G., Chu C., Jiang W., Moorthy B. (2021). Molecular mechanisms of pulmonary carcinogenesis by polycyclic aromatic hydrocarbons (PAHs): Implications for human lung cancer. Semin. Cancer Biol..

[10] Gastelum G., Jiang W., Wang L., Zhou G., Borkar R., Putluri N., Moorthy B. (2020). Polycyclic Aromatic Hydrocarbon-induced Pulmonary Carcinogenesis in Cytochrome P450 (CYP)1A1- and 1A2-Null Mice: Roles of CYP1A1 and CYP1A2. Toxicol. Sci..

[11] Tang D., Phillips D.H., Stampfer M., Mooney L.A., Hsu Y., Cho S., Tsai W.Y., Ma J., Cole K.J., She M.N. (2001). Association between carcinogen-DNA adducts in white blood cells and lung cancer risk in the physicians health study. Cancer Res..

[12] Ceppi M., Munnia A., Cellai F., Bruzzone M., Peluso M.E.M. (2017). Linking the generation of DNA adducts to lung cancer. Toxicology.

[13] Munnia A., Giese R.W., Polvani S., Galli A., Cellai F., Peluso M.E.M. (2017). Bulky DNA Adducts, Tobacco Smoking, Genetic Susceptibility, and Lung Cancer Risk. Adv. Clin. Chem..

[14] Gilberson T., Peluso M.E., Munia A., Lujan-Barroso L., Sanchez M.J., Navarro C., Amiano P., Barricarte A., Quiros J.R., Molina-Montes E. (2014). Aromatic adducts and lung cancer risk in the European Prospective Investigation into Cancer and Nutrition (EPIC) Spanish cohort. Carcinogenesis.

[15] Bak H., Autrup H., Thomsen B.L., Tjonneland A., Overvad K., Vogel U., Raaschou-Nielsen O., Loft S. (2006). Bulky DNA adducts as risk indicator of lung cancer in a Danish case-cohort study. Int. J. Cancer.

[16] Luan Y., Xing G., Ren J., Gu J. (2015). Role of hepatic cytochrome P450 enzymes in the detoxication of aristolochic acid I; effects on DNA adduct, mutation, and tumor formation. Genes Environ..

[17] Hang B. (2004). Repair of exocyclic DNA adducts: Rings of complexity. Bioessays.

[18] Wang L.E., Hu Z., Sturgis E.M., Spitz M.R., Strom S.S., Amos C.I., Guo Z., Qiao Y., Gillenwater A.M., Myers J.N. (2010). Reduced DNA repair capacity for removing tobacco carcinogen-induced DNA adducts contributes to risk of head and neck cancer but not tumor characteristics. Clin. Cancer Res..

[19] Foresta M., Izzotti A., La Maestra S., Micale R., Poggi A., Vecchio D., Frosina G. (2014). Accelerated repair and reduced mutagenicity of DNA damage induced by cigarette smoke in human bronchial cells transfected with *E. coli* formamidopyrimidine DNA glycosylase. PLoS ONE.

[20] Barnes J.L., Zubair M., John K., Poirier M.C., Martin F.L. (2018). Carcinogens and DNA damage. Biochem. Soc. Trans..

[21] Manson M.M., Farmer P.B., Gescher A., Steward W.P. (2005). Innovative agents in cancer prevention. Recent Results Cancer Res. Fortschr. Krebsforsch..

[22] Barhoumi R., Mouneimne Y., Chapkin R.S., Burghardt R.C. (2014). Effects of Fatty Acids on Benzo[a]pyrene Uptake and Metabolism in Human Lung Adenocarcinoma A549 Cells. PLoS ONE.

[23] Zhou G.D., Richardson M., Fazili I.S., Wang J., Donnelly K.C., Wang F., Amendt B., Moorthy B. (2010). Role of retinoic acid in the modulation of benzo(a)pyrene-DNA adducts in human hepatoma cells: Implications for cancer prevention. Toxicol. Appl. Pharmacol..

[24] Piazzi G., D’Argenio G., Prossomariti A., Lembo V., Mazzone G., Candela M., Biagi E., Brigidi P., Vitaglione P., Fogliano V. (2014). Eicosapentaenoic acid free fatty acid prevents and suppresses colonic neoplasia in colitis-associated colorectal cancer acting on Notch signaling and gut microbiota. Int. J. Cancer.

[25] Jing K., Song K.S., Shin S., Kim N., Jeong S., Oh H.R., Park J.H., Seo K.S., Heo J.Y., Han J. (2011). Docosahexaenoic acid induces autophagy through p53/AMPK/mTOR signaling and promotes apoptosis in human cancer cells harboring wild-type p53. Autophagy.

[26] Lim K., Han C., Xu L., Isse K., Demetris A.J., Wu T. (2008). Cyclooxygenase-2-derived prostaglandin E2 activates beta-catenin in human cholangiocarcinoma cells: Evidence for inhibition of these signaling pathways by omega 3 polyunsaturated fatty acids. Cancer Res..

[27] Sun S.Y. (2005). Chemopreventive agent-induced modulation of death receptors. Apoptosis.

[28] Zhou G.D., Zhu H., Phillips T.D., Wang J., Wang S.Z., Wang F., Amendt B.A., Couroucli X.I., Donnelly K.C., Moorthy B. (2011). Effects of dietary fish oil on the depletion of carcinogenic PAH-DNA adduct levels in the liver of B6C3F1 mouse. PLoS ONE.

[29] Mernitz H., Lian F., Smith D.E., Meydani S.N., Wang X.D. (2009). Fish oil supplementation inhibits NNK-induced lung carcinogenesis in the A/J mouse. Nutr. Cancer.

[30] Song E.A., Kim H. (2016). Docosahexaenoic Acid Induces Oxidative DNA Damage and Apoptosis, and Enhances the Chemosensitivity of Cancer Cells. Int. J. Mol. Sci..

[31] Reddy M.V., Randerath K. (1986). Nuclease P1-mediated enhancement of sensitivity of 32P-postlabeling test for structurally diverse DNA adducts. Carcinogenesis.

[32] Zhou G.D., Hernandez N.S., Randerath E., Randerath K. (1999). Acute elevation by short-term dietary restriction or food deprivation of type I I-compound levels in rat liver DNA. Nutr. Cancer.

[33] Gupta R.C. (1984). Nonrandom binding of the carcinogen N-hydroxy-2-acetylaminofluorene to repetitive sequences of rat liver DNA in vivo. Proc. Natl. Acad. Sci. USA.

[34] Randerath K., Reddy M.V., Gupta R.C. (1981). 32P-labeling test for DNA damage. Proc. Natl. Acad. Sci. USA.

[35] Zhu H., Cabrera R.M., Wlodarczyk B.J., Bozinov D., Wang D., Schwartz R.J., Finnell R.H. (2007). Differentially expressed genes in embryonic cardiac tissues of mice lacking Folr1 gene activity. BMC Dev. Biol..

[36] Zhou G.D., Randerath K., Donnelly K.C., Jaiswal A.K. (2004). Effects of NQO1 deficiency on levels of cyclopurines and other oxidative DNA lesions in liver and kidney of young mice. Int. J. Cancer.

[37] Randerath K., Randerath E., Zhou G.D., Supunpong N., He L.Y., McDonald T.J., Donnelly K.C. (1999). Genotoxicity of complex PAH mixtures recovered from contaminated lake sediments as assessed by three different methods. Environ. Mol. Mutagenesis.

[38] Chang W.C., Chapkin R.S., Lupton J.R. (1997). Predictive value of proliferation, differentiation and apoptosis as intermediate markers for colon tumorigenesis. Carcinogenesis.

[39] Zhou G.D., Popovic N., Lupton J.R., Turner N.D., Chapkin R.S., Donnelly K.C. (2005). Tissue-specific attenuation of endogenous DNA I-compounds in rats by carcinogen azoxymethane: Possible role of dietary fish oil in colon cancer prevention. Cancer Epidemiol. Biomark. Prev..

[40] Mabon N., Moorthy B., Randerath E., Randerath K. (1996). Monophosphate 32P-postlabeling assay of DNA adducts from 1,2:3,4-diepoxybutane, the most genotoxic metabolite of 1,3-butadiene: In vitro methodological studies and in vivo dosimetry. Mutat. Res..

[41] Zar J.H. (2009). Biostatistical Analysis.

[42] Randerath E., Zhou G.D., Donnelly K.C., Safe S.H., Randerath K. (1996). DNA damage induced in mouse tissues by organic wood preserving waste extracts as assayed by 32P-postlabeling. Arch. Toxicol..

[43] Miller B.G., Doust E., Cherrie J.W., Hurley J.F. (2013). Lung cancer mortality and exposure to polycyclic aromatic hydrocarbons in British coke oven workers. BMC Public Health.

[44] Phillips D.H. (2002). Smoking-related DNA and protein adducts in human tissues. Carcinogenesis.

[45] Poirier M.C., Beland F.A. (1994). DNA adduct measurements and tumor incidence during chronic carcinogen exposure in rodents. Environ. Health Perspect..

[46] Shahidi F., Ambigaipalan P. (2018). Omega-3 Polyunsaturated Fatty Acids and Their Health Benefits. Annu. Rev. Food Sci. Technol..

[47] Takezaki T., Inoue M., Kataoka H., Ikeda S., Yoshida M., Ohashi Y., Tajima K., Tominaga S. (2003). Diet and lung cancer risk from a 14-year population-based prospective study in Japan: With special reference to fish consumption. Nutr. Cancer.

[48] Vega O.M., Abkenari S., Tong Z., Tedman A., Huerta-Yepez S. (2021). Omega-3 Polyunsaturated Fatty Acids and Lung Cancer: Nutrition or Pharmacology?. Nutr. Cancer.

[49] Lee K.H., Seong H.J., Kim G., Jeong G.H., Kim J.Y., Park H., Jung E., Kronbichler A., Eisenhut M., Stubbs B. (2020). Consumption of Fish and omega-3 Fatty Acids and Cancer Risk: An Umbrella Review of Meta-Analyses of Observational Studies. Adv. Nutr..

[50] Rose D.P., Connolly J.M. (1999). Omega-3 fatty acids as cancer chemopreventive agents. Pharmacol. Ther..

[51] Wendel M., Heller A.R. (2009). Anticancer actions of omega-3 fatty acids--current state and future perspectives. Anti-Cancer Agents Med. Chem..

[52] Zhang G., Panigrahy D., Mahakian L.M., Yang J., Liu J.Y., Stephen Lee K.S., Wettersten H.I., Ulu A., Hu X., Tam S. (2013). Epoxy metabolites of docosahexaenoic acid (DHA) inhibit angiogenesis, tumor growth, and metastasis. Proc. Natl. Acad. Sci. USA.

[53] Hong M.Y., Lupton J.R., Morris J.S., Wang N., Carroll R.J., Davidson L.A., Elder R.H., Chapkin R.S. (2000). Dietary fish oil reduces O6-methylguanine DNA adduct levels in rat colon in part by increasing apoptosis during tumor initiation. Cancer Epidemiol. Biomark. Prev..

[54] Hong M.Y., Bancroft L.K., Turner N.D., Davidson L.A., Murphy M.E., Carroll R.J., Chapkin R.S., Lupton J.R. (2005). Fish oil decreases oxidative DNA damage by enhancing apoptosis in rat colon. Nutr. Cancer.

[55] Jayathilake A.G., Senior P.V., Su X.Q. (2016). Krill oil extract suppresses cell growth and induces apoptosis of human colorectal cancer cells. BMC Complement. Altern. Med..

[56] Notarnicola M., Tutino V., De Nunzio V., Dituri F., Caruso M.G., Giannelli G. (2017). Dietary omega-3 Polyunsaturated Fatty Acids Inhibit Tumor Growth in Transgenic ApcMin/+ Mice, Correlating with CB1 Receptor Up-Regulation. Int. J. Mol. Sci..

[57] Roos P.H., Bolt H.M. (2005). Cytochrome P450 interactions in human cancers: New aspects considering CYP1B1. Expert Opin. Drug Metab. Toxicol..

[58] Thakur V.S., Liang Y.W., Lingappan K., Jiang W., Wang L., Barrios R., Zhou G., Guntupalli B., Shivanna B., Maturu P. (2014). Increased susceptibility to hyperoxic lung injury and alveolar simplification in newborn rats by prenatal administration of benzo[a]pyrene. Toxicol. Lett..

[59] Moorthy B., Miller K.P., Jiang W., Williams E.S., Kondraganti S.R., Ramos K.S. (2003). Role of cytochrome P4501B1 in benzo[a]pyrene bioactivation to DNA-binding metabolites in mouse vascular smooth muscle cells: Evidence from 32P-postlabeling for formation of 3-hydroxybenzo[a]pyrene and benzo[a]pyrene-3,6-quinone as major proximate genotoxic intermediates. J. Pharmacol. Exp. Ther..

[60] Smith M.T., Guyton K.Z., Gibbons C.F., Fritz J.M., Portier C.J., Rusyn I., DeMarini D.M., Caldwell J.C., Kavlock R.J., Lambert P.F. (2016). Key Characteristics of Carcinogens as a Basis for Organizing Data on Mechanisms of Carcinogenesis. Environ. Health Perspect..

[61] Jee S.C., Kim M., Kim K.S., Kim H.S., Sung J.S. (2020). Protective Effects of Myricetin on Benzo[a]pyrene-Induced 8-Hydroxy-2’-Deoxyguanosine and BPDE-DNA Adduct. Antioxidants.

[62] Meital L.T., Windsor M.T., Perissiou M., Schulze K., Magee R., Kuballa A., Golledge J., Bailey T.G., Askew C.D., Russell F.D. (2019). Omega-3 fatty acids decrease oxidative stress and inflammation in macrophages from patients with small abdominal aortic aneurysm. Sci. Rep..

[63] Ali F.F., Rifaai R.A. (2019). Preventive effect of omega-3 fatty acids in a rat model of stress-induced liver injury. J. Cell Physiol..

[64] Wiest E.F., Walsh-Wilcox M.T., Walker M.K. (2017). Omega-3 Polyunsaturated Fatty Acids Protect Against Cigarette Smoke-Induced Oxidative Stress and Vascular Dysfunction. Toxicol. Sci..

[65] Li Z., Pearlman A.H., Hsieh P. (2016). DNA mismatch repair and the DNA damage response. DNA Repair.

[66] Tudek A., Czerwinska J., Kosicki K., Zdzalik-Bielecka D., Shahmoradi Ghahe S., Bazlekowa-Karaban M., Borsuk E.M., Speina E. (2020). DNA damage, repair and the improvement of cancer therapy—A tribute to the life and research of Barbara Tudek. Mutat. Res..

[67] Abbotts R., Wilson D.M. (2017). Coordination of DNA single strand break repair. Free. Radic. Biol. Med..

[68] Uno S., Dalton T.P., Derkenne S., Curran C.P., Miller M.L., Shertzer H.G., Nebert D.W. (2004). Oral exposure to benzo[a]pyrene in the mouse: Detoxication by inducible cytochrome P450 is more important than metabolic activation. Mol. Pharmacol..

[69] Uno S., Dalton T.P., Dragin N., Curran C.P., Derkenne S., Miller M.L., Shertzer H.G., Gonzalez F.J., Nebert D.W. (2006). Oral benzo[a]pyrene in Cyp1 knockout mouse lines: CYP1A1 important in detoxication, CYP1B1 metabolism required for immune damage independent of total-body burden and clearance rate. Mol. Pharmacol..

[70] Shi Z., Dragin N., Galvez-Peralta M., Jorge-Nebert L.F., Miller M.L., Wang B., Nebert D.W. (2010). Organ-specific roles of CYP1A1 during detoxication of dietary benzo[a]pyrene. Mol. Pharmacol..

[71] Boudreau M.D., Sohn K.H., Rhee S.H., Lee S.W., Hunt J.D., Hwang D.H. (2001). Suppression of tumor cell growth both in nude mice and in culture by n-3 polyunsaturated fatty acids: Mediation through cyclooxygenase-independent pathways. Cancer Res..

[72] Pan J., Keffer J., Emami A., Ma X., Lan R., Goldman R., Chung F.L. (2009). Acrolein-derived DNA adduct formation in human colon cancer cells: Its role in apoptosis induction by docosahexaenoic acid. Chem. Res. Toxicol..

[73] Berquin I.M., Min Y., Wu R., Wu J., Perry D., Cline J.M., Thomas M.J., Thornburg T., Kulik G., Smith A. (2007). Modulation of prostate cancer genetic risk by omega-3 and omega-6 fatty acids. J. Clin. Investig..

[74] Strouch M.J., Ding Y., Salabat M.R., Melstrom L.G., Adrian K., Quinn C., Pelham C., Rao S., Adrian T.E., Bentrem D.J. (2009). A High Omega-3 Fatty Acid Diet Mitigates Murine Pancreatic Precancer Development. J. Surg. Res..

[75] Jude S., Roger S., Martel E., Besson P., Richard S., Bougnoux P., Champeroux P., Le Guennec J.Y. (2006). Dietary long-chain omega-3 fatty acids of marine origin: A comparison of their protective effects on coronary heart disease and breast cancers. Prog. Biophys. Mol. Biol..

